# A Spectral Comparison of Jarosites Using Techniques Relevant to the Robotic Exploration of Biosignatures on Mars

**DOI:** 10.3390/life8040061

**Published:** 2018-12-06

**Authors:** Liane Loiselle, Michael A. McCraig, M. Darby Dyar, Richard Léveillé, Sean R. Shieh, Gordon Southam

**Affiliations:** 1 Research School of Earth Sciences, The Australian National University, Acton, ACT 2601, Australia; 2 Centre for Planetary Science and Exploration (CPSX), Department of Earth Sciences, Western University, London, ON N6A 5B7, Canada; sshieh@uwo.ca; 3 Lunar & Planetary Lab, The University of Arizona, AZ 85721, USA; mccraig@lpl.arizona.edu; 4 Department of Astronomy, Mount Holyoke College, South Hadley, MA 01075, USA; mdyar@mtholyoke.edu; 5 Department of Earth and Planetary Science, McGill University, Montreal, QC H3A 0E8, Canada; richard.leveille@mcgill.ca; 6 School of Earth & Environmental Sciences, The University of Queensland, St Lucia, QLD 4072, Australia; g.southam@uq.edu.au

**Keywords:** jarosite, Mars, biosignatures, astrobiology, Rio Tinto, Raman spectroscopy, infrared spectroscopy (IR), visible near-infrared spectroscopy (VNIR), Mössbauer spectroscopy, biomineral

## Abstract

The acidic sulfate-rich waters of the Meridiani Planum region were potentially a habitable environment for iron-oxidizing bacteria on ancient Mars. If life existed in this ancient martian environment, jarosite minerals precipitating in these waters may record evidence of this biological activity. Since the Meridiani jarosite is thermodynamically stable at the martian surface, any biosignatures preserved in the jarosites may be readily available for analysis in the current surface sediments during the ongoing robotic exploration of Mars. However, thermal decomposition experiments indicate that organic compound detection of sediments containing jarosite may be challenging when using pyrolysis experiments; the instrument commonly used to assess organic matter in martian samples. So, here, we assess if the biogenicity of the Meridiani-type jarosites can be determined using complimentary spectroscopic techniques also utilized during the robotic exploration of Mars, including the upcoming ExoMars2020 rover mission. An abiotic jarosite, synthesized following established protocols, and a biological jarosite counterpart, derived from a microbial enrichment culture of Rio Tinto river sediments, were used to compare four spectroscopy techniques employed in the robotic exploration of Mars (Raman spectroscopy, mid-infrared (IR) spectroscopy, visible near-infrared reflectance (VNIR) spectroscopy and Mössbauer spectroscopy) to determine if the complimentary information obtained using these instruments can help elucidate the biological influence of Meridiani-type jarosites. Raman spectral differences might be due to the presence of unreacted reagents in the synthetic spectra and not biological contributions. Reflectance (IR/VNIR) spectra might exhibit minor organic absorption contributions, but are observed in both sample spectra, and do not represent a biosignature. Mössbauer spectra show minor differences in fit parameters that are related to crystal morphology and are unrelated to the biological (i.e., organic) component of the system. Results of this study suggest that the identification of biosignatures in Meridiani-type jarosites using the in situ robotic exploration on Mars may be possible but will be challenging. Our work provides additional insight into extraterrestrial biosignature detection and data interpretation for Mars exploration and indicates that sample return missions are likely required to unequivocally resolve the possible biogenicity of the Meridiani sediments or other jarosite-containing sediments.

## 1. Introduction

Whether life ever existed in ancient environments on Mars is a fundamental, unanswered question that continues to motivate some of the exploration of the planet today (e.g., the search for biosignatures with the ExoMars rover [1]). During the nearly sixty years of robotic exploration of Mars using the analytical instruments on probes, orbiters, and landers, our understanding about the planet has considerably advanced [2]); this includes an increased understanding about martian geology, the physical processes associated with the planetary surface, as well as the surface composition of the planet which includes the presence of potential ancient habitable environments [3]. However, a major restriction in our ability to assess the biogenicity of such putative habitable martian environments, or any biosignatures recorded in the sediments preserved in the martian geologic record, is the lack of accessible materials. As of yet, no samples have been returned to terrestrial laboratories from Mars by manned or unmanned space missions. 

The continued robotic exploration of Mars has revealed that sulfate minerals are ubiquitous on the planet’s surface [4]. Sulfate minerals, like jarosite [(K,H_3_O)Fe_3_(SO_4_)_2_(OH)_6_], are indicators of the paleo-environmental conditions present during their formation (e.g., [5]). The detection of jarosite by the Mars Exploration Rover (MER) *Opportunity* using Mössbauer spectroscopy at Meridiani Planum [6] was a seminal moment in martian planetary exploration. The discovery not only confirmed geochemical predictions about the planet (e.g., [7]), but was also mineralogical evidence that liquid water was present at the martian surface during earlier epochs of the planets’ geologic history [3,6]. Beyond Meridiani Planum, jarosite has since been discovered at several additional locations on Mars including in Gale Crater [8] (the location of the ongoing robotic exploration mission Mars Science Laboratory (MSL) [9]). Jarosite minerals have also been discovered in Mawrth Vallis [10], which is a possible landing site for the European Space Agency (ESA) ExoMars 2020 rover mission [1]. 

Not only was the discovery of the Meridiani jarosite of significant geological importance, it also generated significant astrobiological interest as its occurrence in the Meridiani outcrop revealed a potential (ancient) habitable aqueous environment for acidophilic microorganisms [3,11]. On Earth, jarosite forms during the alteration of volcanic rocks by acidic, sulfur-rich fluids near volcanic vents and in low-temperature acid rock drainage (ARD) environments during the oxidation of sulfide minerals. Terrestrial low temperature ARD systems are habitable environments for acidophilic microorganisms [12]. The Rio Tinto river system in Spain is an ARD system where a diverse community of chemolithotrophic microorganisms, predominantly iron-oxidizing acidophilic bacteria, impart biosignatures and microfossils into the Fe-rich river sediments (see [13,14,15] for a comprehensive description of the Rio Tinto system). Surfaces of cobbles within the river near Berrocal are known to have iron-rich secondary mineral coatings (i.e., jarosite) that contain metabolically active iron-oxidizing bacteria [15,16]. River terrace deposits have been forming at the site for at least 2 myr [15], which provides an opportunity for correlations of biosignatures and morphological fossils noted in modern river sediments to those observed in older river deposits, including preserved biological fabrics within inorganic host material (e.g., [15,17,18]). The Rio Tinto river system is a geochemical and mineralogical analog of the ancient aqueous acidic, sulfate-rich environment of the Meridiani Planum region [13,14,17,18,19,20,21,22]. As the focus of future martian missions shifts towards searching for biosignatures (e.g., the ExoMars 2020 rover [1]), terrestrial analogue studies using samples that contain evidence of life, like the Rio Tinto sediments, can help inform our understanding of how to search for life on Mars by helping to develop strategies for biosignature detection during space exploration missions (e.g., [23]).

Thermodynamic calculations [24] and mineralogical experiments [25] suggest that the Meridiani jarosite is stable at current martian surface conditions. This stability makes the Meridiani jarosite an appealing target for future missions searching for ancient evidence of life on Mars since any chemical, isotopic, or textural properties pertaining to the formation of these Hesperian sulfate minerals may be retained [24] and available for analysis in the current modern sediment, including, if present, biosignatures. However, recent work [26] suggests that jarosite minerals, even when present in minor amounts in a sample, weaken the response for organic molecules when pyrolysis is used as the extraction method in experiments. Jarosite minerals release oxygen during thermal decomposition, making organic compound identification challenging, in particular when the organic content of a sample is low [26]. This is concerning since the most common extraction technique used to search for organic matter in martian surface sediment samples during the in situ robotic exploration of Mars (i.e., pyrolysis experiments [27,28]) may not be able to unequivocally detect organic matter in jarosite samples.

Our ability to successfully search for evidence of Earth-like life in extraterrestrial planetary environments is inherently dependent on our ability to recognize evidence of past or present life in analogous terrestrial samples. Additionally, there are time and resource constraints when conducting in situ experiments during the robotic exploration of planetary bodies and advances in our search for biosignatures on Mars can be made by conducting systematic investigations of martian analogue materials here on Earth (e.g., [25,26,29,30]); and the data obtained from such studies can be used to inform future missions and guide the search for biosignatures in martian sediments (e.g., [26,31]).

Nevertheless, there still remains a lack of astrobiology studies that focus on searching for relevant biosignatures using methods similar to the analytical methods employed during the in situ robotic planetary exploration of Mars (e.g., [1,9]) in martian analogue samples demonstrating high habitable environment potential or biosignature preservation potential (e.g., Rio Tinto river system sediments) that include an abiotic control. Therefore, such studies are particularly timely and needed. For example, unlike on Earth, the evolution of photosynthetic organisms is not expected to have occurred on Mars. Therefore, the detection of photosynthetic molecules, like chlorophyll [32], in Rio Tinto sediments using robotic exploration methods have limited applicability in the assessment of martian biosignatures in the Meridiani sediments. In addition, to be confident in our abilities to establish the biogenicity of unknown materials, it is also important to include abiotic (i.e., no life) controls of these analogue materials in experiments and to examine situations and scenarios where life is absent (i.e., the null hypothesis of astrobiology; [33]). It is important to evaluate the possibility that abiotic signatures can be misinterpreted as biogenic indicators (i.e., false positives; see discussion by [34]); as evidence of biogenicity must unequivocally indicate or be derived from biological life. In this study, we address this gap in the analytical astrobiology research of biosignatures by thoroughly characterizing these possibilities in Meridiani-type jarosites by comparing a biological jarosite with a synthetic (i.e., abiotic) counterpart.

Since recent studies [26] suggest that pyrolysis experiments (e.g., [27,28]) might have difficulties searching for organic compounds in martian sediments that contain jarosite, here we endeavor to explore the possibility of discerning if a biologic signature of a Meridiani-type jarosite can be ascertained (i.e., relative to the abiotic spectral counterpart) using some of the spectroscopic methods employed during the robotic exploration for biosignatures on Mars. A biogenic jarosite and a synthetic counterpart are compared using four spectroscopic techniques that are currently employed on Mars (i.e., Mössbauer spectroscopy; e.g., [6]) or are included on upcoming in situ missions (i.e., Raman spectroscopy [35], mid-infrared (IR) reflectance spectroscopy [36] and visible near-infrared (VNIR) reflectance spectroscopy [37]). Specifically, our spectral comparison will provide insight on our ability to use the spectral data generated during martian missions to find unequivocal signatures indicative of life in jarosites (i.e., relative to (abiotic reference) spectral databases), thus helping to inform future exploration strategies (i.e., sampling decisions, triage protocols) in our continued search for life on Mars.

## 2. Materials and Methods 

### 2.1. Sample Preparation

**Figure 1:**
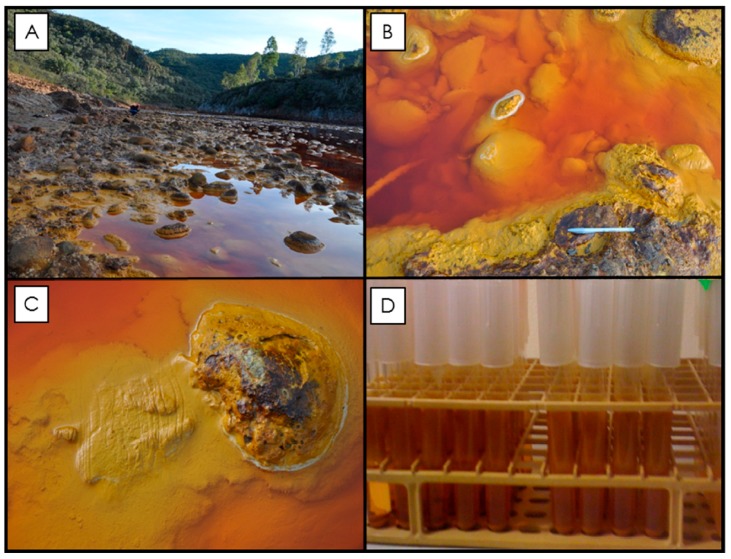
Rio Tinto sampling site and the microbial enrichment culture samples. (**A**) Photograph of the sampling locality near Berrocal, Spain, where samples were taken (37° 35′ 33.27″ N, 6° 33′ 1.84″ W; [38]). The Rio Tinto riverbed is lined with cobbles (see images in **B** and **C**), which, when submerged, are covered with an accumulation of lighter yellow flocculent material. As the cobbles become exposed to more arid conditions, this material dehydrates and becomes a darker ochre color and forms crust precipitates on the river cobbles. These rock coatings are known to contain a consortium of metabolically active iron-oxidizing bacteria [17]). Grab samples were collected from the river (see grooves in **C**) in order to establish a primary culture [38]). Subsequent enrichment cultures were maintained in a laboratory at room temperature and after seven weeks the enrichment culture contained orange precipitates along the side of test tubes and in solution (**D**).

Two samples, a biogenic jarosite sample and a synthetic jarosite sample, are used here. The biogenic material is generated as a by-product of a microbial enrichment culture derived from the Meridiani analogue site Rio Tinto (Figure 1) and the abiotic counterpart is synthesized following an established protocol by precipitating material from a solution at elevated temperature, replicating the jarosite material used in the spectral libraries and databases that identified the mineral on the martian surface (e.g., [6]). By comparing these two end-member jarosite samples (i.e., life and no life), we aim to determine if there are any candidate measurable biosignature observations (or series of observations) that can be uniquely attributed to the biogenicity of the biological sample (e.g., interaction of organics and mixtures, systematic alteration of mineralogical structure, etc.) by the analytical techniques used during the in situ robotic search for biosignatures on Mars. Natural samples are excluded from this study as they are (compositionally) mixtures of unknown amounts of biological and abiotic process.

#### 2.1.1. Biological Sample

The biogenic jarosite sample was generated by using an established enrichment culture of autotrophic, acidophilic, iron-oxidizing bacteria known to precipitate jarosite minerals as a result of their metabolism [17,38,39,40,41,42,43,44,45]. The primary enriched culture was derived from crust precipitates and hydrated mineral samples collected from the Berrocal sampling site of the Rio Tinto river system in Spain (37° 35′ 33.27″ N, 6° 33′ 1.84″ W; [15,16,39]). The primary enrichment culture was made by processing the natural Rio Tinto samples through a series of limiting dilutions in filter-sterilized (0.45 µm pore-sized) modified 9K buffer basal salt growth medium to isolate the dominant bacterial species [38,39,46]. The modified 9K buffer basal salt growth medium contained (per L dH_2_O): 0.1 g K_2_HPO_4_, 0.4 g MgSO_4_·7H_2_O, 0.4 g (NH_4_)_2_SO_4_. Subsequent enrichment cultures were maintained using 4.5 mL of 9K buffer media containing 33.3 g/L FeSO_4_·7H_2_O adjusted to pH 2.2. Filter-sterilized medium was dispensed into autoclaved 13 × 100 mm borosilicate test tubes, inoculated, and covered with plastic push caps to allow free gas diffusion in order to maintain aerobic conditions. Cultures were incubated and left to grow for seven weeks at room temperature (~22 °C) on a laboratory bench. As the bacteria in the culture oxidized the ferrous iron (Fe^2+^) in the culture media, ferric iron (Fe^3+^) hydrolyzed, turning the clear media orange and solid precipitates formed in the solution as well as along the sides of the test tube (see Figure 1D for details). Microbial culture precipitates (i.e., the biogenic sample) were collected from the test tubes on 0.45 µm pore size filters, rinsed with 500 mL ultra-pure water (18 MΩ cm, Milli-Q), and dried at 60 °C for 72 h to remove any adsorbed water molecules. The mineralogical composition and crystal morphology of the fine-grained, powder precipitates were verified with powder X-ray diffraction (XRD) analysis and scanning electron microscopy (SEM) analysis, respectively.

#### 2.1.2. Synthetic Sample

A synthetic counterpart was prepared following the method outlined in [47] by precipitating the minerals from a solution. A 1 L solution containing 30 g of KNO_3_, 8 g of Fe_2_(SO_4_)_3_, and 0.01 M H_2_SO_4_ was heated in open air and continuously stirred for 3 h at 85 °C. The resultant precipitate was scraped from the beaker, filtered using 0.45 µm pore size filters, and rinsed with ~1 L of ultra-pure water (18 MΩ cm, Milli-Q) to remove any soluble ions. The rinsed precipitate was dried for 72 h in an oven at 60 °C to remove any loosely bound water from the mineral precipitate. Powder XRD analysis and SEM of the fine-grained powder sample was used to verify the mineralogical composition of the precipitate and the morphology of the synthesized material, respectively.

#### 2.1.3. X-Ray Diffraction (XRD)

XRD analysis was conducted in order to verify the mineralogical composition of both of the prepared samples. The dried mineral precipitates (see above) were ground in a mortar and pestle to obtain a fine powder and then back-packed into the well of a frosted glass slide to reduce surface roughness and to decrease preferential grain alignment. A Rigaku Rotaflex 180 mm diffractometer with a rotating-anode diffractometer and a graphite-diffracted beam monochromator (Laboratory for Stable Isotope Science, Western University) operating at 45 kV and 160 mA with a cobalt anode source (Co Kα, λ = 1.78897 Å) was used to collect powder X-ray diffraction profiles from 2° to 82° 2θ with a 0.05° step-size and a 30 sec per step counting time. XRD profiles were integrated and analyzed using the Bruker AXS EVA software package [48] and the International Center for Diffraction Data (ICDD) PDF-4 database.

#### 2.1.4. Scanning Electron Microscopy (SEM)

SEM analysis was used to document the morphology of the jarosite mineral samples. Microbial cultures were fixed using glutaraldehyde (2% *v*/*v*) and immobilized onto 0.45 µm filters before being dehydrated using a graded ethanol series (25, 50, 75, and 100% × 3), critical point dried (SamDri^®^-PVT-3B) and mounted onto a 12 mm carbon adhesive tab stuck to an aluminum stub. The synthetic fine-grained powder was pressed directly onto a carbon adhesive tab that was fixed to an aluminum stub. All samples were platinum-coated prior to imaging to reduce charging artifacts. A LEO 1530 Field Emission SEM (The Nanofabrication Facility, Western University) operating with an accelerating voltage of 5.0 kV was used to image the jarosite samples.

### 2.2. Spectroscopy of Jarosite Samples

#### 2.2.1. Raman Spectroscopy

Raman spectra were acquired using 514.5 nm monochromatic light generated by an argon laser source with laser output power controlled at <50 mW, generating a 1–3 µm laser beam. The scattered light was detected using a custom-built laser Raman spectrometer (JASCO NRS 2000; Western University) that was calibrated using neon and silica emission spectra. Raman spectra of biological and synthetic jarosite were collected from 200 to 1600 cm^−1^ (in two parts) and from 3150 to 3650 cm^−1^. Three total accumulations of 60 sec were collected for each region at a spectral resolution of ±2 cm^−1^. Raman spectral band positions were determined using PeakFit and IGOR software suites.

#### 2.2.2. Mid-Infrared Spectroscopy (IR)

Diffuse Reflectance Infrared Fourier Transform Spectra (DRIFTS) were acquired using a Nicolet Nexus 670 FTIR spectrometer (New MIRA Laboratory, Institute of Meteoritics, University of New Mexico) equipped with a Pike AutoDIFF with Globar source, KBr beamsplitter and DTGS detector. Prior to analysis, the system was purged with dry air to decrease atmospheric absorptions (i.e., CO_2_, H_2_O) and care was taken to avoid preferential grain alignment, pitting and/or clumping when placing powders into the AutoDIFF sample cups for analysis. IR data were collected for each sample using 500 scans from 4000 to 400 cm^−1^, at a spectral resolution of 4 cm^−1^. IR band positions were determined using Nicolet OMNIC software.

#### 2.2.3. Visible Near-Infrared Reflectance Spectroscopy (VNIR)

Visible near-infrared reflectance (VNIR) spectra were acquired with an Analytical Spectral Devices Inc. (ASD) FieldSpec Pro HR spectrometer (University of Winnipeg). Measurements were made at a 30° phase angle (i = 30°, e = 0°) using a 50 watt quartz–tungsten–halogen quasi-collimated light source. VNIR spectra of unsorted powders were acquired in absolute reflectance mode from 350 to 2500 nm relative to a Spectralon^®^ standard and 200 individual spectra were averaged to increase the signal-to-noise ratio. Sample spectra were collected with a spectral resolution that varies between 2 and 7 nm (across the bandwidths of each of the instruments’ three detectors) which are then output by the instrument using a proprietary ASD cubic spline function at an interpolated 1 nm spectral resolution, and the spectra were corrected post-output for subtle breaks which often occur at the detector junctions (1000 and 1830 nm) by normalizing the below 1000, and above 1830 nm portions of the spectrum to the central, thermoelectrically cooled detector. VNIR band positions were determined using the Nicolet OMNIC software package.

#### 2.2.4. Mössbauer Spectroscopy

Mössbauer spectra of the two jarosites were collected to characterize the Fe^3+^/Fe^2+^ contents and to examine the polyhedral distortion surrounding those cations. Mössbauer spectra were acquired at room temperature (295 K) using a source of ~40 mCi 57Co in Rh on a WEB Research Co. model WT302 spectrometer (Mount Holyoke College). Sample powders (~10–20 mg) were mixed with sugar for analysis and then mounted in a holder confined by Kapton^®^ polymide film tape. Mössbauer spectra were collected at 295 K over a ±4 mm/s velocity range in 2048 channels and corrected for nonlinearity via interpolation to a linear velocity scale that was defined by the 25 µm Fe foil calibration spectrum. The WMOSS algorithm fits a straight line to the points defined by the published values of the Fe metal peak positions (y values) and the observed positions in channels (x values). Before fitting, the spectrum was folded about the channel value producing the minimum least squares sum difference between the first half of the spectrum and the reflected second half of the spectrum using the WMOSS Auto-fold procedure. All data were corrected to remove the fraction of the baseline due to the Compton scattering of 122 keV gamma rays by electrons inside the detector. The data were modelled using Dist3e software (University of Ghent), which does not presume any particular shape of distribution. Isomer shift (δ) and quadrupole splitting (ΔE_Q_) of the doublets were allowed to vary, while widths of both peaks in each doublet were coupled to vary in unison (i.e., one width was defined for each doublet, but every doublet could vary independently). Errors are ±0.02 mm/s for isomer shift, quadrupole splitting and line widths, with errors of 1–3% (absolute) for the relative area of the distributions (see [49] for detailed discussion about error bars for Mössbauer measurements).

## 3. Results

### 3.1. Samples

**Figure 2:**
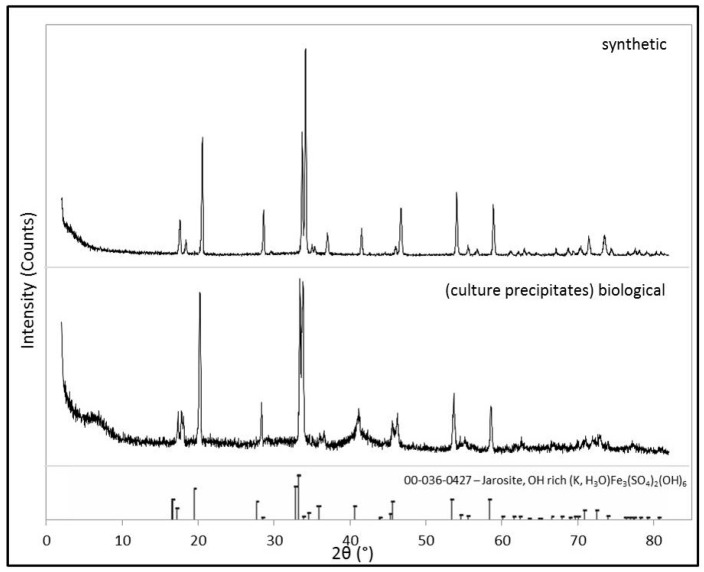
XRD patterns of the synthetic and biological jarosite. Powder X-ray diffraction (XRD) patterns of both the samples prepared in this study for spectroscopic analysis. Mineral identification of the synthesized material and the microbial culture precipitates (i.e., the biological sample) are consistent with the Fe^3+^-bearing sulfate mineral jarosite [(K,H_3_O)Fe_3_(SO_4_)_2_(OH)_6_]. The International Centre for Diffraction Data (ICDD) database card pattern (card no. 036-0427), indicated by the stick pattern at the bottom of the figure, is included for reference).

XRD pattern analysis, presented in Figure 2, verifies the mineralogical compositions of both the biological and synthetic sample as the mineral jarosite ([(K,H_3_O)Fe_3_(SO_4_)_2_(OH_6_)]). All of the peaks observed in each XRD pattern correspond to the d−spacings of jarosite (ICDD card no. 036-0427) and no impurities (e.g., KNO_3_) or secondary phases (e.g., Fe−oxides) are observed in the XRD patterns of either sample. As expected, the XRD pattern of the synthetic jarosite sample exhibits a higher signal-to-noise ratio than that of the biological sample, indicating that the biological jarosite contains a larger amorphous component and is more poorly organized (i.e., less crystalline) than the synthetic counterpart. However, our jarosite samples generate XRD patterns that are in good agreement and broadly consistent with the XRD patterns of natural and synthetic jarosite samples, respectively [15,23,47,50,51,52], and the biological sample pattern is particularly comparable to jarosites derived from other iron-oxidizing bacterial enrichment cultures [38,40,53].

**Figure 3:**
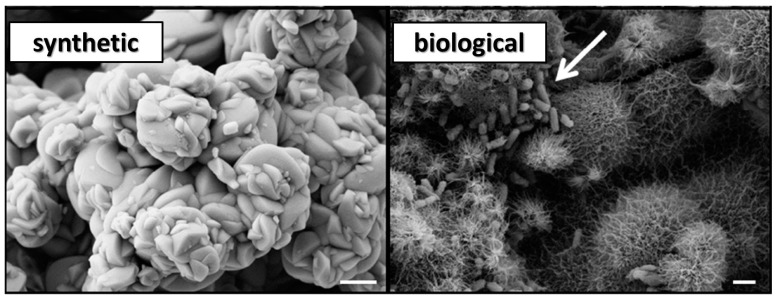
Scanning electron microscope (SEM) images of the synthetic jarosite and the biological jarosite samples. SEM micrographs of the jarosite samples, mineralogical composition confirmed by XRD analysis (see Figure 2), demonstrate a dissimilar mineral morphology at the micron level (scale bar = 1 µm). The synthetic jarosite sample occurs as aggregates of smooth, well-formed crystal surfaces, forming relatively massive rosettes. In contrast, the microbial culture precipitates, herein termed biological jarosite, are radiating aggregates of acicular, needle-like precipitates. Additionally, the presence of rod-shaped bacteria (white arrow) are observed in the biological jarosite sample.

As seen in Figure 3, when the two samples are compared using scanning electron microscopy (SEM), some dissimilarity between the two samples is observed. First, the biological jarosite sample, derived from a microbial enrichment culture of iron oxidizing bacteria, contains distinct biogenic evidence as the rod-shaped bacterial cells and biological fabrics (e.g., EPS; extracellular polymeric substances) within the sample that are readily observed in SEM images (see arrow in Figure 3). EPS are organic macromolecules synthesized by bacteria that are mostly composed of carbohydrates (e.g., polysaccharides) and proteins in addition to other bio macro-molecules (e.g., uronic acids and nucleic acids; [54]). EPS is an important fabric that is secreted by bacteria into the surrounding environment, typically remaining attached to the outer surface of the bacterial cell and is a major constituent of terrestrial biofilms (for a comprehensive review of the role of EPS in geomicrobiology, see [54]).

In addition to the presence of these biological materials, the two compositionally similar jarosite mineral precipitates have noticeably different crystal structures at the microscopic level (Figure 3). The synthetic sample contains no evidence of bacterial cells or biological fabrics (e.g., EPS) and exhibits a morphology of well-formed subhedral, tabular, smooth-faced crystal that forms in closely spaced, radiating rosettes which form blocky, columnar-like chains of aggregates. Whereas, by contrast, the mineral precipitates of the biological sample occur as compact, massive, intergrown anhedral crystals with long, acicular-to-fibrous, needle-like crystal surfaces that radiate uniformly from the center of spherical crystal aggregates; sometimes more broadly described as a ‘hedgehog like’ morphology [47].

### 3.2. Spectroscopy

#### 3.2.1. Raman Spectroscopy

**Figure 4:**
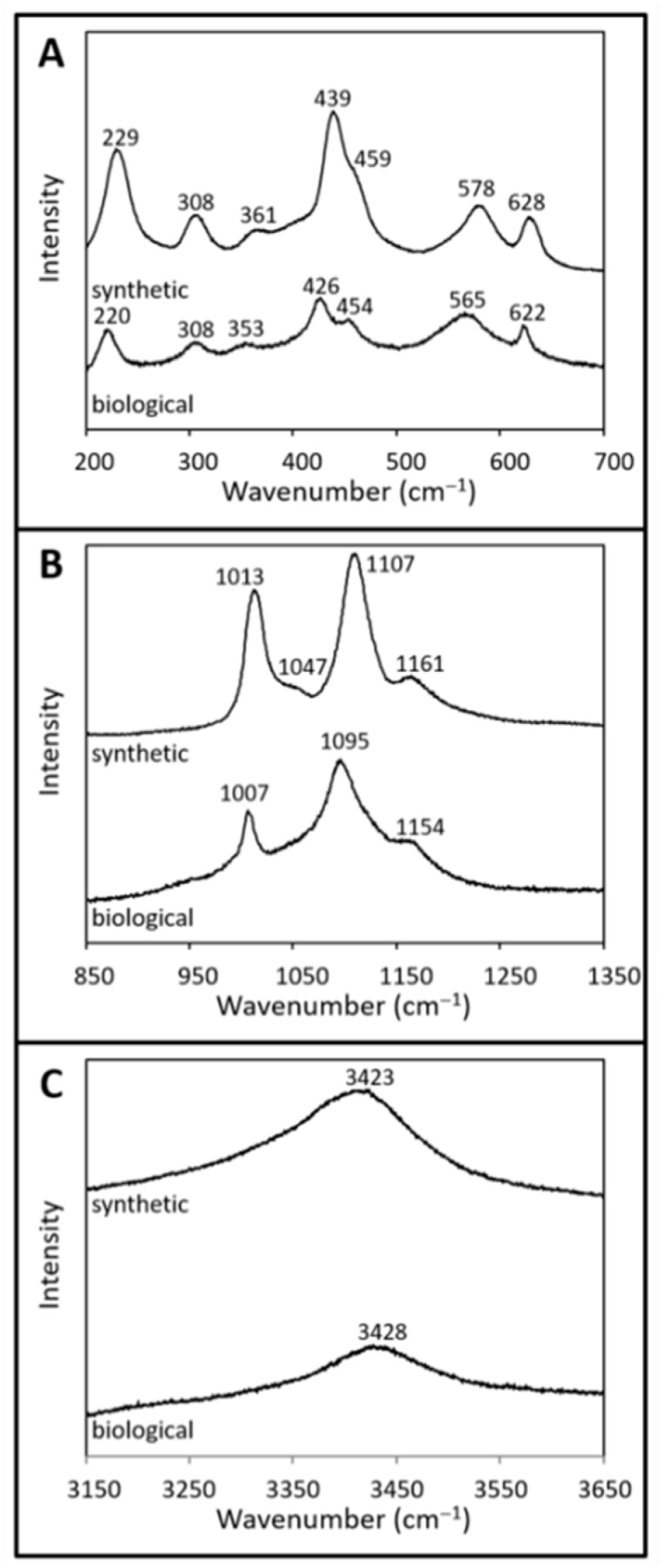
Raman spectra of synthetic and biological jarosite. Raman spectra from (**A**) 200 to 700 cm^−1^, (**B**) 850 to 1350 cm^−1^, and (**C**) 3150 to 3650 cm^−1^ of synthetic jarosite and biological jarosite. The spectral plots are of normalized intensity (arbitrary values) versus wavenumber with a spectral precision of ±2 cm^−1^. Spectra are stacked in order to facilitate comparisons. Peak positions and assignments are listed in Table 1.

Raman spectra of the synthetic and biological jarosite samples from 200 to 700 cm^−1^ (low wavenumber), 850 to 1350 cm^−1^ (mid wavenumber) and from 3150 to 3650 cm^−1^ (high wavenumber) are shown in Figure 4A, 4B, and 4C, respectively. No peaks are observed between 1350 and 1600 cm^−1^ in either spectrum. Raman spectra peak positions and assignments are listed in Table 1 and demonstrate characteristic features expected of jarosite minerals [51,55,56,57,58,59] and correspond well to other Raman spectral analysis of jarosite materials from Rio Tinto (e.g., [23,32]).

The Raman spectrum of the biological sample comprises spectral features related to jarosite minerals which include contributions from the sulfate ion, crystal lattice, and hydroxyl ions (Figure 4, Table 1). The shoulder band at 454 cm^−1^ and the sharp band near 622 cm^−1^ correspond to the symmetric (v_2_(SO_4_^2−^)) and asymmetric (v_4_(SO_4_^2−^)) bending vibrational mode of the sulfate ion, respectively. Likewise, spectral bands at 1095 cm^−1^ and 1154 cm^−1^ are assigned to the asymmetric stretching (v_3_(SO_4_^2−^)) vibration modes, while the peak at 1007 cm^−1^ corresponds to the symmetric (v_1_(SO_4_^2−^)) stretching vibrational mode of the sulfate ion. Four bands in the lattice mode spectra, 426 cm^−1^, 353 cm^−1^, 308 cm^−1^, and 220 cm^−1^, are attributed to the Fe–O vibrational modes in jarosite-group minerals (Figure 4A, Table 1). In the biological spectra, characteristic features of vibrational modes associated with hydroxyl ions (γ(OH)) are observed at 565 cm^−1^ (Figure 4A) and again in the high-wavenumber region at 3428 cm^−1^ as an intense, broad spectral band (Figure 4C).

The Raman spectrum of the synthetic sample is presented in Figure 4 and Table 1, and shows spectral features associated with vibrational modes of the sulfate ion and the hydroxyl ion. Bending vibrational modes of the sulfate ion corresponding to the symmetric (v_2_(SO_4_^2−^)) and asymmetric (v_4_(SO_4_^2−^)) modes occur at 459 cm^−1^ and 628 cm^−1^, respectively (Figure 4A). The asymmetric stretching (v_3_(SO_4_^2−^)) vibrational mode of the sulfate ion corresponds to the bands observed at 1107 cm^−1^ and 1161 cm^−1^, while the symmetric (v_1_(SO_4_^2−^)) stretching mode is attributed to the band at 1013 cm^−1^. Four bands observed in the low wavenumber region (439 cm^−1^, 361 cm^−1^, 308 cm^−1^, and 229 cm^−1^; Figure 4A, Table 1) are attributed to the lattice mode vibrational bands of Fe–O bonds in jarosite-type compounds. Vibrational modes associated with hydroxyl ions (γ(OH)) occur at 578 cm^−1^ and again as a broad band near 3423 cm^−1^ (vOH) in the high wavenumber region (Figure 4C). Additionally, the synthetic jarosite spectrum contains a band in the mid-wavenumber region at 1047 cm^−1^, which is attributed to the strong symmetrical stretching band of the nitrate ion (v_1_(NO_3_); Figure 4B, Table 1). This contribution may stem from some unreacted reagent used during the synthesis of the jarosite sample [55]. 

#### 3.2.2. Mid-Infrared Spectroscopy (IR)

**Figure 5:**
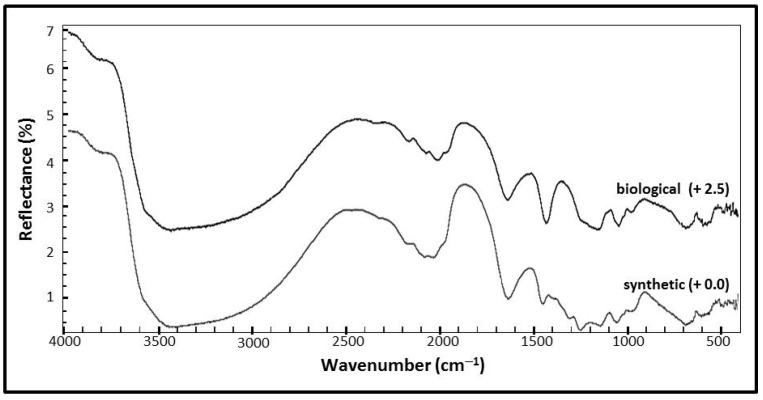
Mid-infrared (IR) spectra of synthetic and biological jarosite. Infrared spectra of biological and synthetic jarosite samples collected from 400 to 4000 cm^−1^. Peak positions and assignments are listed in Table 2. Plots are of percent reflectance versus wavenumber. The spectra are stacked in order to facilitate comparisons with a spectral resolution of 4 cm^−1^.

The Diffuse Reflectance (mid)Infrared Fourier Transform (DRIFTS) spectra of percent reflectance versus wavenumber from 4000 to 400 cm^−1^ of the biological and synthetic samples are shown in Figure 5. The spectral resolution of each spectrum is 4 cm^−1^ and the samples are offset to facilitate comparisons. The IR spectral peak positions and corresponding assignments are listed in Table 2. The spectra exhibit characteristic fundamental vibrations expected of jarosite minerals and are similar to mid-IR spectra obtained for other natural and synthetic jarosite minerals [42,50,59,60,61,62,63,64,65].

Figure 5 shows the mid-infrared (IR) spectrum of the synthetic jarosite sample with the corresponding assignments in Table 2. Spectral features resulting from the fundamental vibrational modes of the sulfate ion occur at 680 cm^−1^, 980 cm^−1^, 1012 cm^−1^, 1053 cm^−1^, 1136 cm^−1^, 1245 cm^−1^ and again as overtones at higher wavenumbers at 1968 cm^−1^, 2031 cm^−1^, 2075 cm^−1^, 2175 cm^−1^, 2322 cm^−1^, and 2460 cm^−1^. The absorption feature at 485 cm^−1^ is assigned to the lattice mode bending vibrational mode of Fe–O, while the spectral contributions related to OH and H_2_O groups occur at 594 cm^−1^, 1634 cm^−1^, 3405 cm^−1^, 3835 cm^−1^, and 3956 cm^−1^. The Christiansen feature might be observed in the synthetic sample spectrum at 1245 cm^−1^ [62].

The mid-infrared (IR) spectrum of the biological jarosite is presented in Figure 5 with corresponding assignments in Table 2. Spectral features related to fundamental vibrational modes of the sulfate ion occur at 733 cm^−1^, 974 cm^−1^, 1058 cm^−1^, and 1187 cm^−1^ with the corresponding overtones occurring in the higher wavenumber region at 1945 cm^−1^, 2025 cm^−1^, 2069 cm^−1^, and 2163 cm^−1^. Four features at the lowest wavenumbers of the spectra (410 cm^−1^, 428 cm^−1^, 461 cm^−1^, and 510 cm^−1^) represent Fe–O lattice mode contributions, while the spectral contributions related to OH and H_2_O groups occur at 556 cm^−1^, 1639 cm^−1^, and 3406 cm^−1^.

#### 3.2.3. Visible Near-Infrared Reflectance Spectroscopy (VNIR)

**Figure 6:**
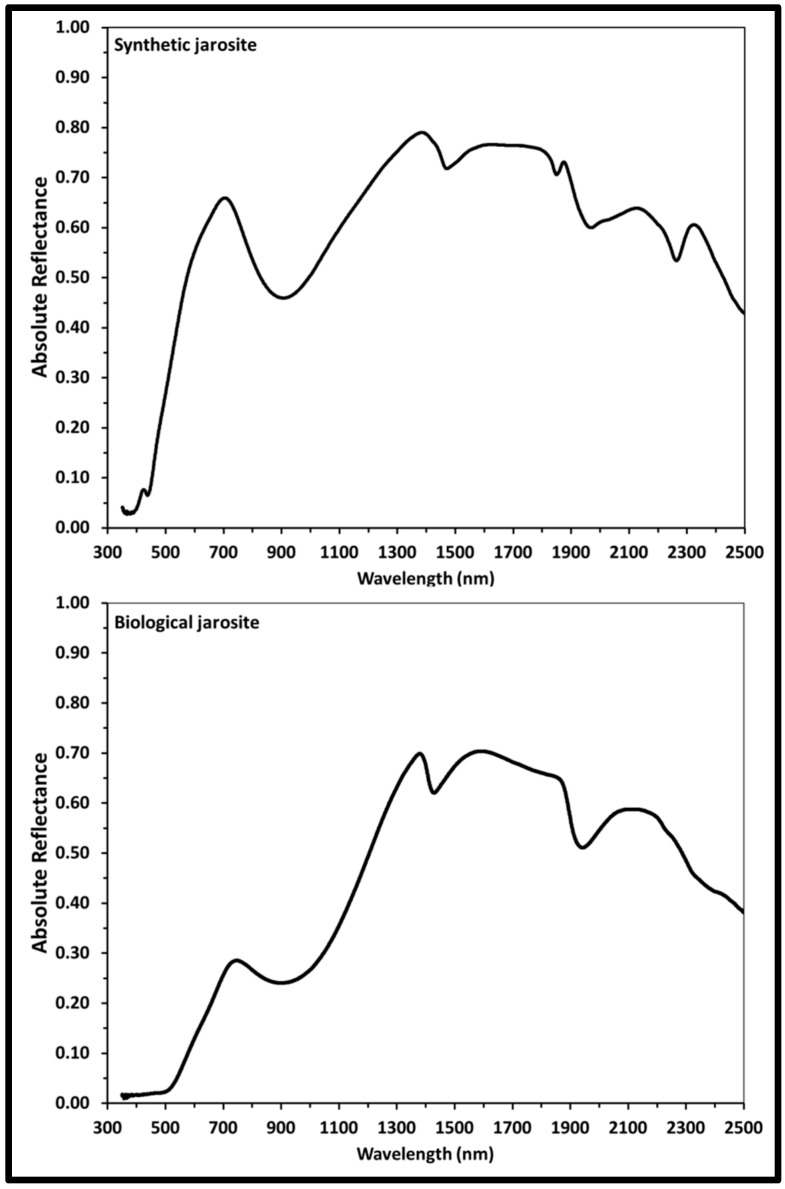
Visible near-infrared reflectance (VNIR) spectra of synthetic and biological jarosite. VNIR reflectance spectra (*i* = 30°, *e* = 0°) of synthetic and biological jarosite minerals obtained from 300 to 2500 nm. Band centers and peak assignments are listed in Table 3. Plots are of absolute reflectance versus wavelength with varying resolution across the spectral range (2 to 7 nm).

The visible near-infrared reflectance (VNIR) spectral plots of absolute reflectance (percentage) versus wavelength from 300 to 2500 nm of the samples are shown in Figure 6. Absorption band centers and the corresponding assignments are listed in Table 3. Both the synthetic and the biological sample exhibit characteristic absorption bands of Fe^3+^-bearing sulfate minerals, including contributions from transition elements (Fe^3+^), hydroxyl (OH), water (H_2_O), and sulfate (SO_4_^2−^) [23,25,60,62,64,66,67].

The VNIR spectrum of the synthetic K-rich jarosite sample (Figure 6, Table 3) exhibits absorption bands due to the Fe^3+^ crystal field transitions at 437 nm, 905 nm and as a weak shoulder centered about 602 nm. Spectra absorption bands due to the vibrational modes of the hydroxyl ion due to OH^−^ overtones at 1470 nm and 2033 nm and due to OH^−^ combinations at 1848 nm and 1969 nm. The absorption feature in the synthetic spectrum at 2263 nm is due to the Fe-O-H vibrational mode combination. 

The VNIR spectrum of the biological jarosite sample (Figure 6, Table 3) exhibits absorption bands due to Fe^3+^ crystal field transitions at 902 nm with a broad shoulder at 506 nm, due to OH^−^ overtones at 1427 nm and 2230 nm, due to OH^−^ combinations at 1941 nm and a series of weaker bands in the longer wavelengths of the spectrum (i.e., above 2300 nm) are due to S−O bending overtones or various OH combinations.

#### 3.2.4. Mössbauer Spectroscopy

**Figure 7:**
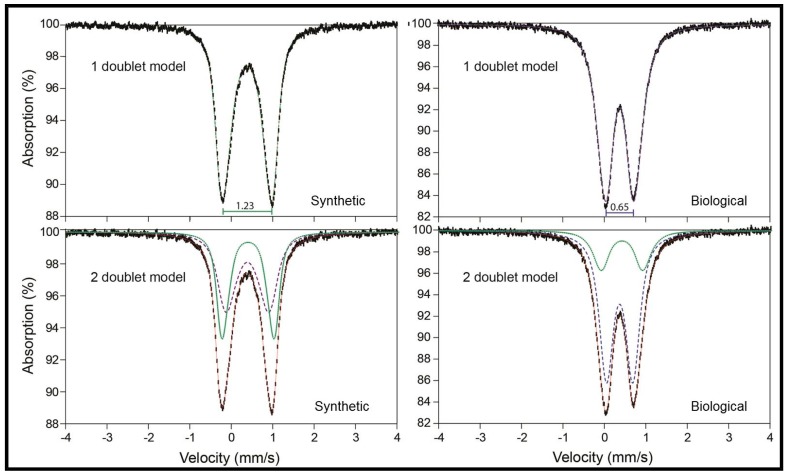
Mössbauer spectra at 295 K of synthetic and biological jarosite. Mössbauer spectra of the synthetic and biological jarosite samples collected over a ±4 mm/s velocity range. Plots are of percent absorption versus velocity with acceptable range errors (±0.02 mm/s). Fit parameters are listed in Table 4. In the one-doublet models, it is apparent that the two jarosite samples are quite different, as indicated by their distinct values of quadrupole splitting. In the two-doublet models, it is shown that both of the spectra can be modeled with two doublets (green dotted line and blue dashed line in bottom spectra), one of which is the same in both (green dotted line), and the fit envelope is shown as a thin red solid line. These data suggest fundamental differences in the Fe^3+^ coordination polyhedra of the two samples.

The Mössbauer spectra of absorption versus velocity collected at 295 K over a ±4 mm/s velocity of the synthetic and biological jarosite samples are shown in Figure 7. The spectra are collected over a ± 4 mm/s range, with errors of ±0.02 mm/s and the red line represents the fit envelope. Spectra are fit with one- and two-doublet models and curve parameters are listed in Table 4. The spectra of both jarosite samples exhibit a characteristic Fe^3+^ double with no evidence of a sextet at room temperature. Fe^2+^ is not observed in either sample. These spectral fit parameters are consistent with Fe^3+^ cations occupying only one octahedral site in the jarosite mineral crystal structure. In the one-doublet model, synthetic and biogenic samples exhibit similar isomer shift (δ) values (0.38 and 0.37 mm/s, respectively) but distinctly different quadrupole splitting (ΔE_Q_) values (1.23 mm/s and 0.65 mm/s, respectively). However, the two-doublet models (Table 4) show that in fact both samples contain Fe^3+^ in a site with ΔE_Q_ = 1.0 ± 0.04 mm/s, while the second doublet has a higher ΔE_Q_ (1.28 mm/s) in the synthetic sample and a lower ΔE_Q_ (0.61 mm/s) in the biogenic sample.

## 4. Discussion

The purpose of this study was to compare an abiotic and biotic jarosite to determine if either sample contained spectroscopic evidence that could potentially be used to assess the biogenicity of the Meridiani-type jarosites during any future in situ robotic exploration of biosignatures on Mars. Spectral comparisons of the synthetic jarosite and the biological jarosite (Figure 4, Figure 5, Figure 6, and Figure 7) indicate that both samples exhibit similar features that are characteristic of jarosite minerals. As there does not appear to be a clear, unequivocal signature indicative of biogenicity in our biological Meridiani-type jarosite when compared to the abiotic synthetic sample, we suggest that discerning the biogenicity of a Meridiani-type jarosite during the in situ robotic exploration of Mars using these spectroscopic techniques may be challenging and sample return may be required in order to unequivocally assess the biogenicity of the Meridiani sediments.

The Raman spectra of both samples demonstrate the characteristic features expected of jarosite minerals, including lattice vibrations and metal–oxygen bonds corresponding to hydroxyl and sulfate bonds, as reported in previous studies of jarosite minerals ([51,55,58,59,60]; Table 1). Spectral comparison of the two jarosites Raman spectra reveals that the synthetic jarosite spectrum exhibits a weak shoulder at 1047 cm^−1^ (Table 1, Figure 2) that is not observed in the corresponding biological sample spectra. A peak at 1047 cm^−1^ is observed in similar jarosite synthesis studies (e.g., [55]) and may correspond to the total symmetric stretching mode of the nitrate ion *v*_1_(NO_3_), from unreacted KNO_3_ reagent during synthetic sample synthesis. However, others have assigned the peak to δOH^−^ [68]. VNIR spectral absorptions of nitrate tend to overlap with those of hydrated sulfate minerals [69], making the spectral identification of the nitrate reagent in the corresponding synthetic (IR/VNIR) spectra difficult. However, we see no evidence of impurities (i.e., potassium nitrate) in the XRD pattern (Figure 2). As this spectral band might be an artifact of the synthesis protocol and is not commonly observed in natural jarosite samples [58,59,60], the absence of this spectral band (~1050 cm^−1^) in Raman spectra does not represent a biosignature that can be used to assess the biogenicity of an unknown jarosite sample. The slight differences in the wavenumber of the observed Raman spectral band positions (i.e., Raman shift) of both samples may be an indication that the biological sample contains more H_3_O^+^ and less K^+^ in the A site of than the synthetic counterpart; this is also demonstrated by our XRD analysis (Figure 2), IR, and VNIR analyses (Figure 5 and Figure 6, respectively; see discussion below). However, the shift observed between the two spectra here are in agreement with the variation of natural and synthetic jarosites that are documented in the literature for these mineral samples (e.g., [51,55,56,59,68]).

Our VNIR and IR reflectance spectra are similar to jarosite spectra generated by previous studies of natural and synthetic jarosite minerals, exhibiting characteristic spectral features associated with the sulfate ion, metal–oxygen bonds and hydroxyl ions.

The VNIR spectra of both jarosite samples (Figure 6) match well to established spectra from previous VNIR studies with the biological VNIR spectra matching well to published VNIR spectra of naturally occurring jarosite and the synthetic VNIR sample spectra of this study in agreement with published synthetic jarosite spectra [25,62,64,66,67]. Unlike the IR spectra, no absorption band contributions in the VNIR spectra are observed that might be attributed to organic contributions. All of the observed VNIR absorption features are consistent with fundamental vibrational modes and overtones/combination bands of jarosite (Table 3, Table 4, Figure 5, Figure 6). As is also observed in the mid-IR spectra (Figure 5), the biological sample generates a VNIR spectrum (Figure 6) with fewer absorption features than in the synthetic counterpart, which is in agreement with previous studies. For example, the absorption feature at ~430 nm in the synthetic spectrum is not observed in the biological spectrum, but not all jarosite spectra show this peak in reflectance spectra (e.g., [62]). The number of absorption bands in a spectrum, the wavelength position of absorption bands and the intensity of these absorption bands can be a function of the composition and structure of the sulfate mineral analyzed. For example, cation substitution can affect the wavenumber positions of all major absorption bands of jarosite reflectance spectra. So, slight differences in the observed absorption band positions between both samples may indicate that the biological sample contains more H_3_O^+^ and less K^+^ in the A site of the jarosite mineral than the synthetic counterpart. This is common for jarosite precipitates generated from enrichment cultures of iron-oxidizing bacteria (e.g., [40]) and is supported by our XRD analysis (Figure 2) and Raman spectra (Figure 4).

Comparison of the IR spectra (Figure 5) indicates that both spectra are similar and the biological spectrum exhibits fewer spectral absorption features than the synthetic counterpart, in agreement with published IR spectra for synthetic and naturally occurring jarosites [42,60,61,62,63,64]. The synthetic spectrum (Figure 5) exhibits more absorption bands due to overtones and combinations of the vibrational modes of the sulfate ion than the biological counterpart, which may pertain to the observed structural differences of the two jarosite samples at the micron level (Figure 2). An additional spectral feature is also observed in the synthetic spectra at 1245 cm^−1^ that may be attributed to the Christiansen feature that occurs in Fe−sulfate reflectance spectra. The Christiansen feature is a reflectance minima observed in reflectance spectra that occurs when the real part of the index of refraction approaches that of the surrounding medium (i.e., dry air) [62,70]. The presence of the Christiansen feature only sometimes occurs in reflectance spectra and is particularly sensitive to the grain size and cementation of a sample [62]. The absence or presence of this feature should not be taken as an indication of the biogenicity of a jarosite sample. Despite being observed in geological samples, the potential of using the presence and position of the Christiansen feature to identify minerals at planetary surfaces might be limited as the feature is not consistently observed in the reflectance spectra of sulfate minerals (e.g., [62,70]).

Biomolecules, derived from the bacterial cells and EPS observed in the SEM images of the biological sample (Figure 3), were anticipated as potential sources of organic material that could generate spectral absorption bands in the biological sample that would not be observed in the synthetic spectra. IR studies focusing on sulfates from Rio Tinto sediments (i.e., natural samples) generate similar IR spectra to our results. Preston et al. [17] also analyzed the IR spectra of jarosites from a microbial culture derived from Rio Tinto waters. While our biological jarosite spectrum (this study, Figure 5) is very similar to the IR spectra of Preston et al. [17], they observed minor organic absorption bands in these materials, suggesting that the detection of organic matter in jarosite minerals using IR spectroscopy might be challenging due in part to the large contribution of the dominance of the sulfate ion vibrational modes and hydroxyl overtones in the spectra.

A minor organic absorption band may be observed in the IR spectra of both samples at 1445 cm^−1^ (synthetic) and 1426 cm^−1^ (biological). This absorption band is not observed in the IR spectra of many natural and synthetic jarosites [60,62,64]. However, it was observed in a study that characterized mineral-rich dried precipitates derived from a microbial enrichment culture of Rio Tinto river water [17], where the absorption band observed at 1428 cm^−1^ is attributed to a vibrational mode of an aliphatic hydrocarbon δ(CH_2_). Preston et al. [17] examined enrichment culture sample precipitates and natural samples searching for organic matter and did not include abiotic controls in their study. While this absorption band represents organic matter (e.g., a methylidene, or a methylene group), our results indicate that it is not a biosignature, as it is observed in both of our jarosite sample spectra (Figure 5, Table 2).

Spectral misinterpretations exist in the geological literature that can propagate errors and misunderstanding in subsequent spectral interpretations. For example, when analyzing microfossils with Raman spectroscopy, hematite is commonly misidentified as disordered *sp^2^* hybridized carbonaceous material when the two materials co-exist in mixed-phase samples (see [34] for a comprehensive discussion). Analytical astrobiology studies that assess our ability to detect evidence of life in terrestrial analogue samples using instrumentation associated with the exploration of planetary surfaces should aim to address the ‘no life’ scenario by including abiotic, analytical controls. When not compared to an abiotic control, it becomes difficult to assess if any observed potential biosignatures can be generated by an equivalent, or even unknown, abiotic process. For example, Preston et al. [17] characterized biomolecules preserved in natural Rio Tinto sediments, and argue that they possibly observe minor organic absorption bands in the IR spectrum of their mineral-rich dried jarosite precipitate, derived from a microbial enrichment culture, at 1428 cm^−1^ (methylene) and 1644 cm^−1^ (H_2_O, C=C bond or amide I). However, here, we attribute the IR spectral band at ~1640 cm^−1^, (1639 cm^−1^ and 1634 cm^−1^ in the biological and synthetic IR spectra, respectively, in this study) to the δ(H_2_O) vibrational mode of the jarosite mineral, in agreement with the majority of IR spectral studies of jarosites (e.g., [60,61,63] including those on synthetic (i.e., abiotic) jarosites [50,64]). As the 1600cm^−1^ spectral feature is observed in both of our jarosite IR spectra (this study), the spectral absorption feature is commonly observed in IR spectroscopic studies of jarosite that are synthetically made (i.e., no life; e.g., [64]) and any corresponding organic bands related to alkenes and amide bands are not observed in the Raman spectra of our samples, we prefer assigning the spectral contribution in our spectra at ~1600 cm^−1^ to the inorganic δ(H_2_O) vibrational mode instead of an organic absorption band.

Mössbauer spectroscopy is used to identify iron-bearing mineral phases (e.g., oxides, silicates, carbonates, sulfides, and sulfates) in martian rocks and sediments. The technique quantitatively measures the relative distributions of Fe^3+^/Fe^2+^ iron-bearing phases and examines the distortion of the polyhedron surrounding those cations; it does not detect organic matter nor is it intended to directly aid in the search for biological signatures of life within samples. In this study, we employed Mössbauer spectroscopy to investigate whether structural differences are observed in jarosites that form biologically versus synthetically. 

The Mössbauer fit parameters of the two jarosites here (Table 4) are similar to values obtained in situ with the MER *Opportunity* Mössbauer instrument. Klingelhöfer et al. [6] observed an anhydrous sulfate with parameters most consistent with a potassium–sodium jarosite with some aluminum substitution in the B site (δ = 0.39 mm/s, ΔE_Q_ = 1.22 mm/s) at the Eagle Crater outcrop in Meridiani Planum. Additionally, they characterized a secondary, non-mineral specific, octahedrally coordinated, Fe^3+^ doublet with an identical isomer shift but with a lower quadrupole splitting value (ΔE_Q_ = 0.64 mm/s), suggesting that it could result from minerals such as akaganéite, ferrihydrite, superparamagnetic hematite, goethite, lepidocrocite, some phyllosilicates, and/or schwertmannite.

Mössbauer spectra of the two jarosite samples in this study yield distinct fit parameters (Figure 7 fit parameters listed in Table 4). Despite being different, a note of caution is required here, as these differences between the two samples do not represent a biosignature. Spectra of the synthetic and biological jarosite samples yield identical isomer shift values in both one-doublet and two-doublet models. When only one doublet was fit, the synthetic material generates a quadrupole splitting of ΔE_Q_ = 1.23 mm/s that correlates with high octahedral distortion as observed by previous studies of natural and synthetic jarosites [15,61,71]. The biological sample yields a lower splitting value (ΔE_Q_ = 0.65 mm/s) that is significantly lower than expected for jarosite materials [64,72] if a one-doublet model is employed. The hyperfine parameters of the biological jarosite closely match those of schwertmannite [64,72,73,74]. However, two-doublet models produce more consistent results, and both include the dominant jarosite doublet at δ = 0.39 mm/s and ΔE_Q_ = 0.96 and 1.04 mm/s in biological and synthetic jarosite, respectively, but the second doublet is distinctive. In the biological material, the spectrum is dominated by Fe^3+^ in a lower ΔE_Q_ doublet indicative of a more symmetrical polyhedron. Differences in the ΔE_Q_ Mössbauer parameter values are the result of differences in coordination polyhedra geometries surrounding the Fe cation sites in a mineral (e.g., octahedral; see [75] for a comprehensive discussion). Distortions in these surrounding site geometries may be related to the same steric factors controlling the crystal morphologies of minerals and not necessarily indicate any biological influence. Furthermore, similar coordination polyhedra geometries can occur for a number of minerals, making it difficult to unambiguously identify minerals with Mössbauer spectroscopy in the absence of corroborating data. For example, Dyar et al. [75] show that the same Mössbauer parameters attributed to jarosite at Meridiani Planum on Mars [6] can also be exhibited by several other Fe^3+^-bearing sulfates with very similar Fe coordination polyhedra, including botryogen, metasideronatrite, as well as slavikite. The iron-oxyhydroxysulfate mineral schwertmannite also has similar hyperfine parameters to the biological jarosite in this study (Figure 3). Abiogenic and biogenically formed schwertmannite minerals demonstrate radially oriented filamentous crystal morphologies [73,74,76] that are akin to the crystal morphologies of the biological jarosite sample. Nor are Mössbauer fit parameters diagnostic of iron sulfate minerals in particular. For example, many phosphate minerals have Mössbauer spectra quite similar to those of sulfates due to their analogous crystal structures [77]. In fact, the range of hyperfine parameters for Fe^3+^ in rock-forming silicates, oxides, sulfates, phosphates, etc. is very small, and thus is unlikely to be diagnostic of any particular mineral species except in carefully characterized samples. Thus, because it is not possible to unambiguously differentiate a biogenic and inorganic material with similar coordination polyhedra geometry fit parameters (e.g., biological jarosite of this study, inorganic Fe^3+^-bearing sulfates, abiotic or biogenic schwertmannite), the differences in Mössbauer parameters observed for the biological jarosite and the synthetic jarosite sample are unlikely to confirm evidence of biological influence during the continued robotic exploration of Mars.

A growing body of evidence suggests that the detection of any potentially preserved biosignatures in the Meridiani-type jarosites using in situ robotic exploration might be difficult using current martian planetary exploration instrumentation. For example, if the labels are removed and both samples here are treated as unknowns, it becomes very difficult to assign biogenicity or identify biological influence in either sample, using the four spectroscopic methods (Raman, IR, VNIR, and Mössbauer). Results of our spectroscopic comparison indicate that even in well-known terrestrial samples containing biological fabrics (e.g., bacterial cells, EPS) it can be difficult to unequivocally identify biosignatures using techniques employed during the in situ robotic martian exploration missions. Moreover, our results agree with recent investigations assessing the organic matter detection in jarosites using pyrolysis experiments similar to the Sample Analysis at Mars (SAM) instrumentation [9,27]). Lewis et al. [26] demonstrate that jarosite minerals release substantial amounts of oxygen during pyrolysis experiments, complicating assessments of the martian organic record within jarosite samples recovered from the martian surface. Despite these difficulties, the martian jarosites should remain a high-priority target for sample return mission where assessments of mineralogical biosignatures can be made in terrestrial laboratories, not constrained by time, and where methods and analytical tools are constantly improving.

## 5. Conclusions

The Meridiani jarosite is of significant astrobiological interest as it formed in acidic, aqueous conditions that were potentially habitable for acidophilic microorganisms. Here, using a well-characterized terrestrial martian analogue material known to contain life, we demonstrate that assessing evidence of biogenicity in Meridiani-type jarosites (from an abiotic counterpart) using the spectroscopic instruments employed during the continued in situ robotic exploration of Mars may be challenging. 

Raman, IR, VNIR, and Mössbauer spectral comparisons of the two jarosites indicate that both samples exhibit similar spectral features characteristic of jarosite minerals. Raman spectral differences may be due to the presence of unreacted reagents in the synthetic spectra. As the absence of this Raman band is also noted in naturally occurring (abiotic) jarosite samples, it cannot be used to assess the biogenicity of the jarosites on Mars. The VNIR spectra of both samples are similar and the slight spectral differences observed between the two samples agree with variations exhibited by natural jarosite minerals in established VNIR spectroscopy studies. IR spectroscopic results contain possible evidence of minor organic absorption contributions; however, as they are observed in both the biological and synthetic spectra, they do not represent an unequivocal biologic signature. The Mössbauer spectra of the two samples show minor differences in fit parameters that are related to crystal morphology and are unlikely to be related to the biological component of the system.

As there does not appear to be a clear, unequivocal signature indicative of biogenicity in our biological Meridiani-type jarosite, discerning the unequivocal possibility of biologic signatures in jarosites (relative to the synthetic spectra in databases) during the in situ robotic exploration of Mars using these spectroscopic techniques may be challenging.

Despite our inability to confidently distinguish biogenicity using these spectroscopic techniques, our results do not preclude Meridiani sediments remaining high-priority targets in the search for life on Mars. While more work is required to explore the potential biologic signatures retained in this (putative) martian biomineral (from their abiotic mineral counterparts), our contribution continues to highlight the challenges of searching for unambiguous evidence of biosignatures using rover-based exploration strategies. However, the absence of (unequivocal) evidence of biologic signatures in martian samples during in situ robotic investigations should not be taken as evidence that martian sediments are devoid of discernable evidence of life; as such evidence might be available for discovery, for example, in returned samples or during manned exploration missions to Mars.

## Figures and Tables

**Table 1:** Raman spectra band assignments of the fundamental modes observed in the synthetic and biological jarosite.

Sample	νOH	ν_3_	ν_1_ (NO_3_)	ν_1_	ν_4_	γ(OH)	ν_2_	Fe−O
Synthetic jarosite	3423	1161	1047	1013	628	578	459	439
		1107						361
								308
								229
Biological jarosite	3428	1154		1007	622	565	454	426
		1095						353
								308
								220

Raman spectral bands observed in the spectra (Figure 4) collected from 200 to 3650 cm^−1^ and the corresponding assignments-based published values [23,32,51,55,56,58,59].

**Table 2:** IR spectral band positions and assignments.

Reflectance Spectra (all bands in cm^−1^)		
Synthetic	Biological	Assignment
3956		3ν_3_ (SO_4_)^2−^, OH OT/C
3835		3ν_3_ (SO_4_)^2−^, OH OT/C
3405	3406	ν (OH)
2460		
2322		2ν_3_ (SO_4_)^2−^, 2 δ (OH)
2175	2163	2ν_3_ (SO_4_)^2−^, 2 δ (OH)
2075	2069	2ν_3_ (SO_4_)^2−^, 2 δ (OH)
2031	2025	2ν_3_ (SO_4_)^2−^, 2 δ (OH)
1968	1945	2ν_3_ (SO_4_)^2−^, 2 δ (OH)
1634	1639	δ (H_2_O)
1445	1426	2ν_3_ (SO_4_)^2−^, δ (CH_2_O)
1401		
1301		
1245		Christiansen feature
	1187	ν_3_ (SO_4_)^2−^
1136		δ (OH)
1053	1058	δ (OH)
1012		δ (OH)
980	974	ν_1_ (SO_4_)^2−^
680	733	ν_4_ (SO_4_)^2−^
594	556	γ (OH)
	510	Fe-O
485	461	Fe-O
	428	Fe-O
	410	Fe-O

OT/C denotes overtones and combination bands. IR spectra of samples were collected from 400 to 4000 cm^−1^ (Figure 5). Band assignments are based on published values from [58,62,64,65].

**Table 3:** VNIR spectra band positions and assignments of the fundamental modes observed in the synthetic and biological jarosite.

Reflectance spectra (all band locations in nm)		
Synthetic	Biological	Assignment
437		Fe^3+^ crystal field transition
602 ^a^	506 ^a^	Fe^3+^ crystal field transition
905	902	Fe^3+^ crystal field transition
1470	1427	OH overtones
1848		Combinations of OH or H_2_O bending, stretching, and rotational fundamentals
1969	1941	Combinations of OH or H_2_O bending, stretching, and rotational fundamentals
2033 ^a^	2230 ^a^	*v*OH/H_2_O + γ/δ OH/H_2_O
2263		Fe –O–H combination
	2319 ^a^	3*v_3_*S-O or OH/H_2_O combination and overtone
	2351 ^a^	3*v_3_*S-O or OH/H_2_O combination and overtone

^a^ denotes a weak absorption band or shoulder. VNIR spectra of samples were collected continuously from 300 to 2500 nm (Figure 6) and band assignments are based on published values from [23,25,60,62,64,66,67].

**Table 4:** Mössbauer spectra (295 K) curve fit parameters of synthetic and biological jarosite.

	Sample	δ	Δ	area	δ	Δ	area
One-doublet model							
	Synthetic	0.38	1.23	100			
	Biological	0.37	0.65	100			
Two-doublet model							
	Synthetic	0.39	1.04	53	0.37	1.28	47
	Biological	0.39	0.96	23	0.37	0.61	77

Isomer shift (δ) and quadrupole splitting (Δ) are given in mm/s and area is given as percentage of the total spectra. Error bars are ±0.02 mm/s for δ and Δ and ±1–3% on doublet areas. Mössbauer fit parameters for the octahedrally coordinated Fe^3+^ in synthetic and biological jarosite for the spectra plotted in Figure 7.
